# Supplementary material for: Chromothripsis during telomere crisis is independent of NHEJ, and consistent with a replicative origin
Source: Genome Res. 2019 May;29(5):737–49. doi: 10.1101/gr.240705.118 (PMC6499312; doi:10.1101/gr.240705.118)
Supplement: Supplemental Material [file supp_gr.240705.118_Supplemental_file_1.zip › contigs/annotated_contigs/DB45/contig.2.DB45_length_374_mean_cov_8.94385026738.docx]

**DB45_length_374_mean_cov_8.94385026738**

```
AGCATCCTCATCACCCCCACCCGGGCTCCACCCCCTTGCCTGGGGCCCTCTGGCCGGGCCTGGGAGGCACCTGCTGCAGCTGGCACAGC
 >chr8:142425884-142426207 - E=1e-175
CCCAGGCCTTTCGGAGAAGTCTATTTATAACCAGGCAGGAGCCGTTCCTGGAATCCTCCCAGAGCGGGCCCAAGTCCGCCCATTGCTAG

GGGGCCAGGAGGGGAGACCCAAGCAGGCCAAGCCCACGCTTGATGCACTAGGACGCCTTCCCGGAGTCCTCCAGGCCCCTGCACTCGGC

AGCTCCAAGGGCCCAGCCTCCTCACTGCCACATCCCCCCATGCCATCCCCT|CCCC|CCCCCCCCCCCCCCCCCCCCCCCCCCCCCCCC
                                                    >chr2:33141317-33141373 - E=3e-22
CCCCCCCCCCCCCCCCCCGC
```
